# The Association of Delayed Milk Ejection with Milking Performance in Holstein Cows in a Large Dairy Herd with Suboptimal Premilking Teat Stimulation

**DOI:** 10.3390/ani14121828

**Published:** 2024-06-20

**Authors:** Ajay Singh, Madeleine Eve Spellman, Haritha Somula, Mohammad Osamah Dahl, Matthias Wieland

**Affiliations:** 1 Department of Population Medicine and Diagnostic Sciences, College of Veterinary Medicine, Cornell University, Ithaca, NY 14853, USA; as3527@cornell.edu (A.S.);; 2 Department of Internal and Preventive Medicine, College of Veterinary Medicine, University of Mosul, Mosul 41002, Iraq

**Keywords:** bovine, bimodal, milk ejection, oxytocin

## Abstract

**Simple Summary:**

Here, we investigated the associations of delayed milk ejection with the average milk flow rate, milking duration, and duration in a low milk flow rate in cows in a large dairy herd with suboptimal premilking stimulation. Our second aim was to study the association between peak lactation milk yield and delayed milk ejection. We found that delayed milk ejection was associated with a decreased average milk flow rate, increased milking duration, and increased duration in a low milk flow rate. Further, we found an association between peak lactation milk yield and delayed milk ejection such that cows with lower peak lactation milk yield had greater odds of delayed milk ejection. Our data suggest that delayed milk ejection has a negative impact on milking performance, and that peak lactation milk yield may serve as a proxy for the risk of delayed milk ejection. Future research is warranted to test if delayed milk ejection can be mitigated through, for example, a modified milking routine regimen, and if such a modified regimen has the potential to improve the milking performance in dairy cows.

**Abstract:**

The primary objective was to investigate the association between delayed milk ejection (DME) and the average milk flow rate, milking unit-on time, and duration in a low milk flow rate in Holstein dairy cows in a large dairy herd with suboptimal premilking teat stimulation. Our second objective was to study the association between peak lactation milk yield and the occurrence of DME. This longitudinal field study was conducted at a 4300-cow dairy farm with a thrice-daily milking schedule over a 1-week period. We analyzed data from 61,677 cow milking observations from 2937 cows. Delayed milk ejection was defined as present if the 30–60 s milk flow rate was ≤3.1 kg/min. The mean average milk flow rate (MAMF, kg/min), mean milking unit-on time (MMUT, s), and mean duration of a low milk flow rate (MLMF, s) were calculated as the mean values from the 21 milking observations. General linear multivariable models revealed associations of DME with MAMF, MMUT, and MLMF. A multivariable ordinal logistic regression model revealed an association between peak lactation milk yield and DME. Cows with lower peak lactation milk yield had greater odds of exhibiting a higher frequency level of DME. The observed associations between DME and milking performance indices suggest that DME can negatively affect milking and parlor efficiency. Peak lactation milk yield may serve as a proxy to estimate cows’ risk of recurrent DME. Future research is warranted to test if alleviating DME through, for example, a modified milking routine influences the milking performance indices described herein.

## 1. Introduction

Adequate premilking teat stimulation is critical to facilitate an efficient milk harvest and minimize the impact of the milking machine on the teat tissue condition [1,2]. Premilking teat stimulation is composed of the tactile stimulation of the teat and the timing of the milking unit attachment [3]. The tactile stimulation of the teat activates pressure-sensitive receptors and initiates the release of oxytocin into the bloodstream by the pituitary gland [4]. The lag time between the beginning of tactile stimulation and the attachment of the milking unit allows this hormone to travel through the bloodstream, reach the mammary gland, and bind to myoepithelial cell receptors. The subsequent activation of the myoepithelial cells leads to their contraction and the expulsion of the alveolar milk into the duct system of the mammary gland, making it available for harvesting [5]. 

To meet the physiological needs of a cow, there must be both sufficient tactile stimulation to bring oxytocin levels to about three times the baseline concentration and enough lag time to allow the alveolar milk to be ejected into the gland cistern before the cisternal milk has been evacuated [6]. The milk flow curve of a properly stimulated cow is characterized by a steep incline phase that progresses uninterruptedly into the plateau phase [7]. Conversely, if a premilking stimulation regimen fails to meet a cow’s physiological requirements or the oxytocin release is inhibited, for example, by milking in unfamiliar surroundings [6], the milk ejection is ‘disturbed’ or ‘delayed’ [5,7]. Delayed milk ejection (DME) most often manifests as a bimodal milk flow curve, which is characterized by an increasing milk flow rate that is followed by a decreasing flow rate during the first two minutes of milking [8]. To a lesser extent, DME can occur in the absence of bimodality if only small amounts of cisternal milk are available, for example, after short milking intervals [9]. 

Several studies have investigated the association between DME and milking performance. A field study involving 2486 cow milking observations from 82 Italian Holstein-Friesian dairy herds showed that inappropriate premilking stimulation increased the occurrence of bimodality and decreased milk yield [10]. In a recent observational study conducted on a 3600-cow dairy farm in Michigan, USA, Erskine et al. (2019) examined milking data from 663 Holstein cows milked three times per day [11]. They found that cows with DME yielded 1.8–3.1 kg less milk per milking session than cows without DME [11]. In a study conducted at five dairy farms with a thrice-daily milking schedule in New York, USA [12], our group analyzed data from 241 cow milking observations and revealed that cows with a bimodal milk flow curve produced 1.3 kg less milk per milking session than those with a unimodal milk flow curve. Bruckmaier and Blum used 12 cows to study the effect of premilking stimulation on milk ejection and milk flow [4]. They found that cows that did not receive premilking stimulation exhibited bimodal milk flow and an increased milking unit-on time, although no significant decrease in milk yield was observed [4]. Although these studies have shown that DME can have a negative impact on milking performance in dairy cows, this evidence is limited. For instance, the assessment of the association between DME and milking performance in previous studies was based on a single milking observation [4,11,12]. However, some cows may exhibit DME in every milking session, i.e., chronic DME [13], and the effect size of the association of DME with milking performance could differ according to the frequency of DME events.

Several factors have been associated with an increased occurrence of DME. Inadequate tactile stimulation, improper preparation lag time, milking in unfamiliar surroundings, and short milking intervals have been identified among the external factors associated with DME [6,14,15,16]. To ensure sufficient teat stimulation, the current standard recommends applying tactile stimulation for 15 s [2,17,18], followed by a preparation lag time of 60 to 120 s [3]. In larger dairy herds, the adoption of a milking routine with adequate premilking teat stimulation is rare in practice. This is likely due to the expanding herd size and increased reliance on hired labor [19,20], which necessitates a greater focus on increasing parlor throughput [16]. Sandrucci et al. (2007) reported that as farm size grows, less time is spent on teat stimulation, therefore lowering the overall time for udder preparation [10]. Moore-Foster et al. showed that in larger herds (≥300 cows), the total stimulation duration was reduced by half compared to that in smaller herds [16]. Previous work from our group has shown that the preparation lag time in large dairy operations can be as short as 57 s [12]. 

Cow characteristics that have been related with DME are parity [2,13], the stage of lactation [10], the degree of udder filling [14,17], and health events such as lameness [13] or mastitis [21]. Conversely, to the best of our knowledge, the association between peak lactation milk yield and DME has not been investigated by rigorous methods. 

Traditionally, DME has been assessed by measuring continuous milk flow with electronic milk flow meters such as the Lactocorder (WMB AG, Balgach, Switzerland). With the advancement of on-farm milk flow meter technology that facilitates measurements of milk flow dynamics during each milking session daily, precise measurements of milk flow dynamics are possible. In a recent study, we demonstrated the suitability of using the incremental milk flow rate between 30 and 60 s of milking for the detection of DME [22]. This provides an opportunity to monitor cows’ milk ejection on dairy operations over time. Data from such serial assessment could further our understanding of the association between DME and milking performance among cows with different frequencies of DME and allow us to explore the relationship between cow characteristics (e.g., peak lactation milk yield) and DME. 

The primary objective of this study was to investigate the association between DME and milking performance in Holstein cows in a large dairy herd with a thrice-daily milking schedule over a 1-week period. An extension of our primary objective was to investigate the interrelationship between cow characteristics such as parity and the stage of lactation, DME, and milking performance. We hypothesized that DME would be associated with a lower average milk flow rate, longer milking unit-on time, and longer duration of a low milk flow rate, and that the observed associations would be modified by the effects of parity and the stage of lactation. Our second objective was to study the association between peak lactation milk yield and DME. We hypothesized that cows with a higher peak lactation milk yield were less likely to exhibit DME.

## 2. Materials and Methods

This longitudinal study was conducted at a commercial dairy farm near Ithaca, NY, USA. Some of the data (564 cow observations) were previously used in a case–control study investigating risk factors for DME [13].

### 2.1. Animals and Housing

During the study period, the herd consisted of approximately 4300 lactating Holstein cows housed in free-stall pens bedded with manure solids. Cows were fed a total mixed ration formulated to meet the requirements outlined by the National Research Council (2001). The rolling herd key performance indicators were a projected 305 d mature equivalent milk production of 13,765 kg, rolling mean test day somatic cell count (SCC) of 230,000 cells/mL, monthly clinical mastitis incidence of 7.8%, 21 d pregnancy rate of 29.0%, and culling rate of 36.9%. Herd records were maintained in a dairy management software program (Dairy Comp 305, Valley Agricultural Software, Tulare, CA, USA, https://vas.com/). The farm used Dairy Herd Improvement Association services, including the individual-cow SCC option.

### 2.2. Milking System and Routine

The milking system and routine have been described previously [13]. Briefly, cows were milked three times daily in a 100-stall parallel rotary parlor (RP3100HD, DeLaval International AB, Tumba, Sweden). The vacuum pump (22.4 kW; 30 HP) was set to supply a receiver–operator vacuum of 44 kPa (13.0 inHg). The milking unit was composed of the cluster MC70 (DeLaval International AB, Tumba, Sweden) with a round barrel milking liner (LS-01 NC, DeLaval International AB, Tumba, Sweden). The pulsators (EP100, DeLaval International AB, Tumba, Sweden) operated at a pulsation rate of 60 cycles/min and a ratio of 65:35 and provided side-to-side alternating pulsation. The average claw vacuum during the peak milk flow period (36 kPa) was calculated from ten milking observations using a digital vacuum recorder (VPR100, DeLaval International AB, Tumba, Sweden) according to the guidelines outlined by the National Mastitis Council (2012). The automatic cluster removers were set to a cluster remover milk flow threshold of 1.6 kg/min, a 1 s delay, and a vacuum decay time of 1.5 s. The milk sweep started 3.0 s after the retraction of the milking unit and lasted for 8 s. The rotational speed of the milking parlor was 5.3 s/stall (rotation time: 530 s), resulting in a theoretical throughput of 679 cows/h. Four teat spray robots (TSR, DeLaval International AB, Tumba, Sweden), two at the parlor entrance and two at the parlor exit, were installed for the application of the pre- and postmilking teat dip. Assuming a cow entered the rotary parlor in stall 1, the positioning of the pre-dip teat spray robots was as follows: robot 1, stall 3 and robot 2, stall 5.

The parlor was operated by four milking technicians in two 12 h milking shifts. The milking technicians were assigned to perform the following tasks at four different stations: At station 1, positioned at stall 10, the teat barrel of all teats from lactating quarters was wiped with an individual clean cloth towel. Station 2 was located at stall 11, and the teat ends were wiped with an individual clean cloth towel there. At station 3, positioned at stall 22, the milking unit was attached and aligned. The milking technician at station 4 was positioned at the halfway point of the rotary and operated within a wider range to monitor milking liner slips, unit fall-offs, and unit kick-offs, and to realign or reattach the milking unit accordingly. This set up resulted in a dip contact time of 27–37 s, a duration of tactile stimulation (calculated as the sum of wiping duration of stations 1 and 2) of approximately 6 s, and a preparation lag time (i.e., time from first tactile stimulus to milking unit attachment) of approximately 64 s.

### 2.3. Data Acquisition and Processing 

We used Dairy Comp 305 (Valley Agricultural software) to acquire data on the parity, stage of lactation, SCC, presence or absence of a non-lactating quarter, and peak lactation milk yield (kg). Milking characteristics were assessed at each milking with electronic on-farm flow-through milk meters using near-infrared technology (MM27BC, DeLaval International AB, Tumba, Sweden) and recorded with the dairy farm management software program (DelPro 5.1, DeLaval International AB, Tumba, Sweden). We retrieved the following milking characteristics: the milk yield (amount of milk harvested from start of milking to detachment of the milking unit; kg); 2 min milk yield (amount of milk harvested within the first two min of milking; kg); milk flow rates in the first 15 s, and in the first 15–30 s, 30–60 s, and 60–120 s of milking; average milk flow rate (milk yield/milking unit-on time; kg/min); milking unit-on time (time from the start of milking to the detachment of the milking unit; s); and duration of a low milk flow rate (duration with a milk flow rate < 1 kg/min between start of milking and detachment of the milking unit; s). Data for each milking session were exported as a comma-separated values (csv) file once daily.

### 2.4. Study Design

The study period for this investigation lasted from 22 September to 28 September 2019. All lactating cows during this period were considered the source population. Animals with complete records for all 21 milking observations were considered the study population. A period of one week was selected because it reflected the study farm’s weekly management schedule, including a review of the milking routine protocol.

### 2.5. Data Processing

We compiled the data in Microsoft 365 Excel (Microsoft Corp., Redmond, WA, USA) and JMP Pro 16.0 (2021 SAS Institute Inc., Cary, NC, USA). First, the 1-week milking characteristics reports were merged into a single file, which contained a total of 89,487 milking observations from 4361 cows considered for inclusion in the analyses. In the next step, we identified missing or erroneous values. We excluded 5970 (6.7%) milking observations with missing values of the milking characteristics (multiple enumeration possible) milk yield (*n* = 4851), milking unit-on time (*n* = 4806), average milk flow rate (*n* = 5467), and 2 min milk yield (*n* = 5827), resulting in 83,517 observations from 4176 cows. Last, we excluded 1239 cows that were missing one or more milking observations. The remaining 61,677 milking observations from 2937 cows were complete and included in the final analyses. In the next step, we created a variable for the explanatory variable of interest (i.e., DME) based on the study by Wieland and Sipka [22]. In brief, we dichotomized the data into the presence or absence of DME (i.e., DME present if 30–60 s milk flow rate ≤ 3.1 kg/min; DME absent if 30–60 s milk flow rate > 3.1 kg/min) and calculated the frequency distribution of DME for each cow to create a continuous variable with values ranging from 0 to 21 DME events. In addition, for the outcomes of interest, we created the following variables from the mean values of the 21 milking observations: (1) the mean average milk flow rate (MAMF; kg/min), (2) mean milking unit-on time (MMUT; s), and (3) mean duration of a low milk flow rate (MLMF; s). Further, we created a new variable by categorizing the continuous variable peak lactation milk yield into 4 levels based on the quartiles < 44.5, 44.5–51.6, 51.7–58.5, and >58.5 kg/d. Last, we created an ordinal variable (DME_Q_) by categorizing the continuous variable DME into 4 levels as follows: DME_Q_-0, 0 DME events; DME_Q_-7, 1–7 DME events; DME_Q_-14, 8–14 DME events; and DME_Q_-21, 15–21 DME events.

### 2.6. Statistical Analysis

We performed all statistical analyses using SAS 9.4 (SAS Institute Inc., Cary, NC, USA) and created the figures using GraphPad Prism 10.1.2 (GraphPad Software, San Diego, CA, USA).

#### 2.6.1. DME and Milking Performance

To study the association between DME and milking performance, we fitted 3 multivariable linear regression models with PROC GLM. The dependent variables were MAMF, MMUT, and MLMF. The explanatory variable of interest (i.e., the frequency of DME events) was forced into each model. The initial screening included univariable linear regression analyses, where the parity (1st, 2nd, and ≥3rd), stage of lactation (early, 1–100; mid-, 101–200; and late lactation, >200 DIM), SCC (log10-transformed, logSCC), and presence or absence of a non-lactating quarter were included as independent variables and tested individually. In addition, average milk yield per milking session (kg/milking session) was also tested as an independent variable in modeling the outcome variables MMUT and MLMF. All variables with a *p*-value < 0.20 in the univariable analysis were considered in the initial multivariable models. We assessed the collinearity among eligible variables by calculating Spearman correlation coefficients with PROC CORR. We considered a coefficient > |0.70| to indicate collinearity. In the multivariable analysis, variables with a *p*-value < 0.05 were retained in the models. Biologically relevant two-way interactions between DME and the remaining variables were tested one at a time and retained if the *p*-value < 0.05. We assessed the assumptions of homoscedasticity and normality of residuals by the inspection of residual plots versus corresponding predicted values and examination of quantile–quantile residual plots. To satisfy these assumptions, values of the outcome variable MLMF were log10-transformed. The resulting least squares means (LSMs) were consequently back-transformed and presented as geometric means with 95% confidence intervals (95% CIs). To demonstrate differences in the outcome variables among cows with different frequencies of DME events, we calculated the LSM for hypothetical cows with 0, 7, 14, and 21 milking observations with DME using the ‘at’ function and the ‘lsmeans’ statement. For the outcome variables MMUT and MLMF, calculations were made over the average milk yield per milking session for each level of parity and stage of lactation, respectively.

#### 2.6.2. Peak Lactation Milk Yield and DME

To study the association between peak lactation milk yield and DME, we fitted an ordinal logistic regression model with PROC LOGISTIC. The dependent variable was the ordinal variable DME_Q_ (0, 1–7, 8–14, and 15–21 DME events). We used the ‘descending’ statement, so that the modeled probabilities were cumulated over the higher ordered values (i.e., 15–21 DME events). The explanatory variable of interest, peak lactation milk yield (<44.5, 44.5–51.6, 51.7–58.5, >58.5 kg/d), was forced into the model. The parity, stage of lactation, presence or absence of a non-lactating quarter, and logSCC were the independent variables and screened for inclusion in the initial model through univariable analyses. Variables that led to a *p*-value < 0.20 were considered eligible for inclusion. Spearman correlation coefficients were calculated to test for collinearity among eligible covariates and a coefficient of ≥|0.70| was regarded as the threshold value. To reach the final model, manual backwards selection was performed until all variables had a *p*-value < 0.05. We used the score test to test the proportional odds assumption. Last, we calculated the predicted probabilities of the 4 different DME_Q_ levels for hypothetical cows in parity 2, between 101 and 200 DIM, four lactating quarters, a logSCC of 4.80, and peak lactation milk yields of <44.5, 44.5–51.6, 51.7–58.5, and >58.5 kg/d.

## 3. Results

### 3.1. Descriptive Statistics

**Figure 1:**
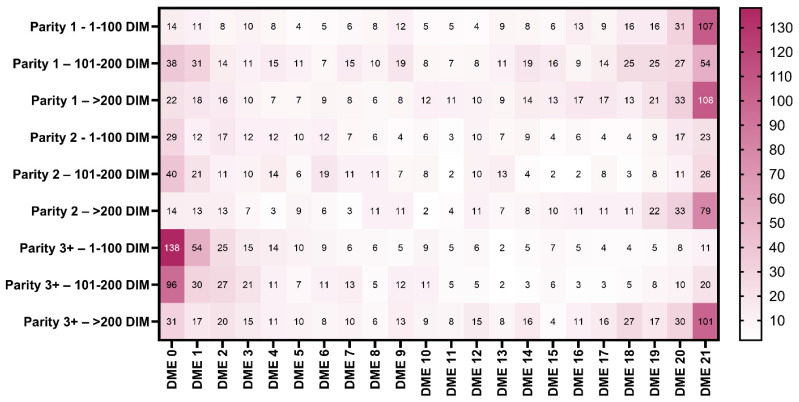
Heatmap showing the frequency distribution of cows with DME events (i.e., 0 to 21 DME events) over a period of 1 week (i.e., 21 milking observations), stratified by parity and the stage of lactation.

Complete records for 21 milking observations were available from 2937/4361 cows (67.3%); thus, a total of 61,677 cow milking observations were included in the final analyses. Cows were in their first (1098, 37.3%), second (769, 26.2%), or ≥third parity (1070, 36.4%). The mean (±standard deviation, SD) DIM on the day of inclusion in this study was 170 ± 103 d. The median (first and third quartiles) SCC in milk from the most recent DHIA test prior to this study was 45,000 (21,000, 119,000) cells/mL. The mean logSCC (±SD) was 4.80 ± 0.57. Among the included cows, 458 (15.6%) had a non-lactating quarter. The mean (±SD) values of milking characteristics calculated from the 61,677 observations were the milk yield, 13.7 ± 3.8 kg/milking session; 2 min milk yield, 6.9 ± 2.3 kg/milking session; first 15 s milk flow rate, 1.0 ± 0.5 kg/min; 15–30 s milk flow rate, 3.0 ± 1.3 kg/min; 30–60 s milk flow rate, 3.1 ± 1.6 kg/min; 60–120 s milk flow rate, 4.3 ± 1.3 kg/min; average milk flow rate, 3.4 ± 0.8 kg/min; milking unit-on time, 242 ± 53 s; and duration of a low milk flow rate, 16 ± 17 s. The mean (±SD) values for MAMF, MMUT, and MLMF were 3.4 ± 0.8 kg/min, 242 ± 47 s, and 16 ± 11 s, respectively. We observed DME in 31,574/61,677 (51.2%) cow milking observations. A total of 529 (18%) cows exhibited DME in every milking session, 422 (14%) cows showed no DME, and 1986 (67.6%) cows exhibited DME events in 1 to 20 milking observations (25th percentile: 2 DME events; 50th percentile: 11 DME events; and 75th percentile: 19 DME events). The distribution of the ordinal variable DME_Q_ was as follows: DME-0, 422 (14.4%); DME-7, 803 (27.3%); DME-14, 513 (17.5%); and DME-21, 1199 (40.8%). The frequency distribution of DME events (i.e., 0 to 21) stratified by parity and the stage of lactation is shown in Figure 1.

### 3.2. DME and Milking Performance

**Figure 2:**
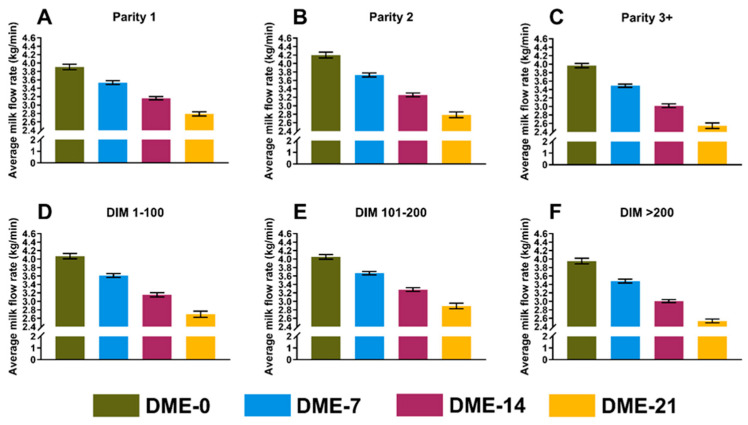
Least squares means from a general linear model showing the mean average milk flow rate (kg/min) of hypothetical cows with 0, 7, 14, or 21 milking observations with DME stratified by parity (**A**–**C**) and the stage of lactation (**D**–**F**). Error bars represent the 95% CI.

**Figure 3:**
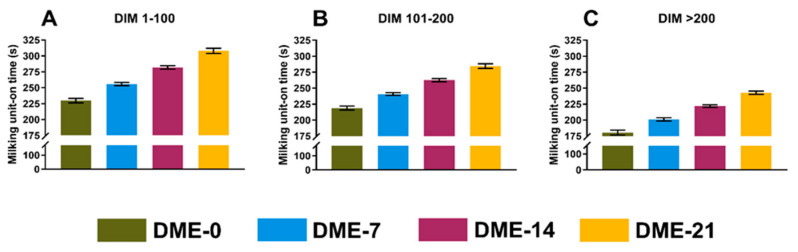
Least squares means from a general linear model showing the average milking unit-on time (s) of hypothetical cows with 0, 7, 14, or 21 milking observations with DME stratified by the stage of lactation (**A**–**C**). Error bars represent the 95% CI.

**Figure 4:**
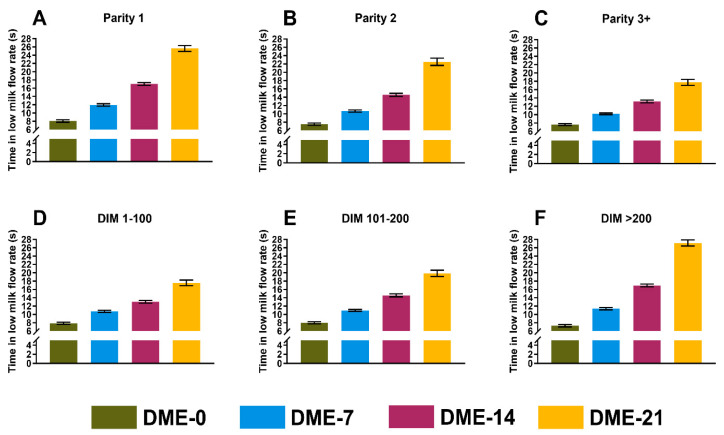
Least squares means from a general linear model showing the average duration of a low milk flow rate (s) of hypothetical cows with 0, 7, 14, or 21 milking observations with DME stratified by parity (**A**–**C**) and the stage of lactation (**D**–**F**). Error bars represent the 95% CI.

The association of DME with milking performance was evaluated based on the three outcome variables MAMF (kg/min), MMUT (s), and MLMF (s). We detected no collinearity among eligible independent variables (r ≤ |0.49|) and thus, all variables were included in the initial models. The results (LSM, 95% CI) from the final models are presented in Table 1, illustrated in Figure 2, Figure 3 and Figure 4, and presented individually below.

#### 3.2.1. Mean Average Milk Flow Rate

The final model included the parity (*p* < 0.0001), stage of lactation (*p* = 0.02), presence or absence of a non-lactating quarter (*p* < 0.0001), logSCC (*p* < 0.0001), DME (*p* < 0.0001), and interactions between DME and parity (*p* < 0.0001) and between DME and the stage of lactation (*p* = 0.0001). The LSMs (95% CI) for cows with and without a non-lactating quarter, respectively, were 3.3 (3.2–3.3) and 3.4 (3.4–3.5) kg/min. A 1-unit increase in logSCC decreased the MAMF by 0.1 (−0.1, −0.1) kg/min. Figure 2 shows the LSM (95% CI) for MAMF stratified by parity and the stage of lactation. The coefficient of determination (R^2^) of the final model was 0.54. 

#### 3.2.2. Mean Milking Unit-On Time

All independent variables included in the initial multivariable model yielded *p*-values ≤ 0.046 and thus were retained. The final model included the parity (*p* < 0.0001), stage of lactation (*p* = 0.007), presence or absence of a non-lactating quarter (*p* = 0.02), logSCC (*p* = 0.046), average milk yield per milking session (*p* < 0.0001), DME (*p* < 0.0001), and interaction between DME and the stage of lactation (*p* < 0.0001). The MMUT (LSM, 95% CI) was 238 (236–240) for cows in parity 1, 238 (236–240) for cows in parity 2, and 252 (251–255) for cows in parity 3 and greater. The presence of a non-lactating quarter increased the MMUT by 3 (0–7) s. The LSMs (95% CI) for cows with and without a non-lactating quarter, respectively, were 245 (242–248) and 241 (240–243) s. A 1-unit increase in logSCC decreased the MMUT by 2 (−4, 0) s. Last, a 1 kg increase in milk yield increased the MMUT by 10 (10–11) s. Figure 3 shows LSMs (95% CI) for MMUT stratified by the stage of lactation. The coefficient of determination (R^2^) of the final model was 0.63.

#### 3.2.3. Mean Duration of Low Milk Flow Rate

As independent variables, the presence or absence of a non-lactating quarter and logSCC revealed *p* ≥ 0.14 in the initial model and thus were excluded. The final model included the parity (*p* = 0.04), stage of lactation (*p* < 0.0001), average milk yield per milking session (*p* < 0.0001), DME (*p* < 0.0001), and interactions between DME and parity (*p* < 0.0001) and between DME and the stage of lactation (*p* = 0.0001). A 1 kg increase in milk yield increased the MLMF by 1 (1–1) s. Figure 4 shows LSMs (95% CI) for MLMF stratified by parity and the stage of lactation. The coefficient of determination (R^2^) of the final model was 0.66. 

### 3.3. Peak Lactation Milk Yield and DME

**Figure 5:**
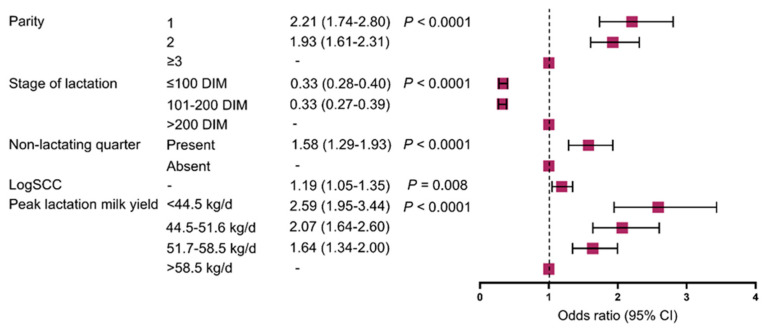
Results from multivariable ordinal logistic regression model showing association of parity, stage of lactation, presence of non-lactating quarter, somatic cell count (log10-transformed, logSCC), and peak lactation milk yield with delayed milk ejection.

All independent variables tested for inclusion in the initial model were eligible (*p* ≤ 0.003). We detected no collinearity among eligible variables, r ≤ |0.67|, and thus, all variables were included in the initial multivariable model and retained (*p* ≤ 0.003). The final ordinal logistic regression model included the parity (*p* < 0.0001), stage of lactation (*p* < 0.0001), presence or absence of a non-lactating quarter (*p* < 0.0001), logSCC (*p* = 0.008), and peak lactation milk yield (*p* < 0.0001) (Figure 5). Cows in parities 1 and 2 had greater odds of a higher DME_Q_ level (i.e., DME-7, DME-14, or DME-21) compared with cows in parity ≥ 3 [odds ratio (OR), 95% CI; parity 1, 2.21 (1.74–2.80); parity 2, 1.93 (1.61–2.31)]. Early and mid-lactation animals had lower odds of having a higher DME_Q_ level [1–100 DIM, 0.33 (0.28–0.40); 101–200 DIM, 0.33 (0.27–0.39)] compared with late-lactation animals. Cows with a non-lactating quarter had greater odds of having a higher DME_Q_ level than those with four lactating quarters [1.58 (1.29–1.93)]. A 1-unit increase in logSCC increased the odds of having a higher DME_Q_ level [1.19 (1.05–1.35)]. Cows with lower peak lactation milk yield had greater odds of a higher DME_Q_ level than those with a peak lactation milk yield of >58.5 kg/d [<44.5 kg/d, 2.59 (1.95–3.44); 44.5–51.6 kg/d, 2.07 (1.64–2.60); and 51.7–58.5 kg/d, 1.64 (1.34–2.00)]. The predicted probabilities of the four different DME_Q_ levels for a hypothetical cow in parity 2, between 101 and 200 DIM, four lactating quarters, a logSCC of 4.80, and the four different levels of peak lactation milk yields are shown in Table 2.

## 4. Discussion

The objective of this study was to investigate the association of DME with milking performance in Holstein dairy cows in a large dairy herd with suboptimal premilking teat stimulation. To carry out this, we used seven days of existing data from electronic on-farm milk flow meters to identify if DME was associated with various milking performance indices. To our knowledge, our study is the first to assess the association of DME with milking performance in a longitudinal study design. This study design allowed us to evaluate the outcome variables over time instead of being limited to a single milking observation, which can reveal more trends associated with DME, such as cows that have chronically delayed milk ejection. The negative impacts of DME, including damage to teat tissue and reduced milking performance, have been reported previously [12,13,14]. Our data add to the existing literature and suggest that DME has a negative impact on MMUT, MLMF, and MAMF.

We found DME in 51.2% of milking observations, which compares to previously documented frequencies of 45.6% [11], 53% [10], and 24% [12]. We found that DME was also associated with parity and days in milk, with cows in parity 1 or 2 having a greater risk of being at a higher DME_Q_ level than the cows in the third or a greater parity, and cows with >200 DIM having a greater risk of an increased DME_Q_ than cows with ≤200 DIM. Additionally, peak lactation milk yield was negatively associated with DME, meaning that cows with lower milk yields at peak lactation were more likely to have a higher DME_Q_. Increased DME was associated with lower MAMF, higher MMUT, and higher MLMF for all cows. In addition to their association with the occurrence of DME, parity and the stage of lactation modified the effect size that DME has on milking performance. These effects were not changed to the same degree by the same stage of lactation or parity though, which points to the different physiological causes of the negative interactions between DME and our chosen milking performance indices. For example, the difference in MMUT between two cows at 100 DIM with 0 or 21 DME events, respectively, was 78 s, while the difference between a pair at 101–200 DIM would be 66 s, and the difference for two cows at >200 DIM would be 63 s. However, the trend of less days in milk being associated with larger effect sizes is reversed with MLMF, where cows later in lactation were more affected by increased numbers of DME events. MAMF, which is a function of milk yield and milking unit-on time, was the most complex in terms of how the stage of lactation modified the effect of DME. In this case, cows in mid-lactation (101–200 DIM) showed the smallest effect of DME with a difference of 1.2 kg/min between cows with 0 and 21 DME events, respectively, while cows between 1 and 100 DIM showed a difference of 1.4 kg/min and cows > 200 DIM showed a difference of 1.5 kg/min. While cows in earlier parities had a higher risk of a higher DME_Q_, they did not always show the greatest effect sizes. Between a cow with 0 DME events and a cow with 21 events, the biggest MAMF difference of 1.4 kg/min would be seen with a pair of cows in parity ≥ 3, while first-parity cows would show a difference of 1.1 kg/min. 

We believe that the high frequency of milking observations with DME can be attributed mostly to the suboptimal premilking teat stimulation conducted at the study farm. Kaskous and Bruckmaier suggested that a stimulation duration of 15 s prior to a latency period of 45 s resulting in a preparation lag time of 60 s is necessary to achieve adequate milk ejection in mid-lactation cows [17]. For cows with very low udder filling, the researchers suggested a stimulation time of 30 s followed by a latency period of 60 s (i.e., preparation lag time of 90 s). Similarly, Weiss and Bruckmaier recommended a premilking stimulation duration of 20 to 90 s based on the degree of udder filling [15]. In the current study, the approximate stimulation and preparation lag times were 6 and 64 s, respectively. This may not have been sufficient to achieve adequate milk ejection, thus leading to high rates of DME. Additionally, the manual forestripping step during premilking stimulation was omitted during this study, which could also explain the high prevalence of DME.

In our previous studies [1,23], we also observed lower odds of DME in multiparous cows compared to primiparous ones, attributing this to the larger teat and gland cistern size in multiparous cows, as reported by other authors [24,25,26]. The increased odds of DME in late-lactation cows (>200 DIM) may be attributable to a reduced level of udder filling during this stage. This finding is supported by previous studies [10,17,24]. Kaskous and Bruckmaier observed a higher proportion of delayed milk ejection (DME) in cows with less alveolar filling of milk before milking compared to cows with more alveolar filling [17]. Sandrucci et al. reported a greater percentage of DME in late-lactation animals (>150 DIM) [10].

Our data indicated that cows with lower peak lactation milk yields have higher odds of DME. We speculate that the observed association can be attributed mostly to differences in the anatomy of the mammary gland among cows with different peak lactation milk yields. That is, we believe that cows with higher peak lactation milk yield have larger gland cisterns than cows with lower peak lactation milk yield. Larger gland cisterns in cows with higher peak lactation milk yields could have facilitated the storage of more milk, which in turn could have taken longer to evacuate, thereby bridging the lag time between the exhaustion of the cisternal milk and the ejection of the alveolar milk. This phenomenon was likely aggravated by the suboptimal premilking teat stimulation. However, because we did not assess mammary gland size, this possible explanation remains speculation. 

The association we observed between MMUT and DME is of particular importance due to the potential costs accrued by a longer unit on time. This correlation is also attested in the literature, with Bruckmaier and Blum [4] theorizing that the longer milking unit-on time seen with DME is due to a delay in the availability of the alveolar milk fraction, resulting in an interruption of milk flow, as well as a transfer of the milking vacuum into the mammary gland during the low milk flow period. Additionally, the increased MMUT associated with DME can have a substantial financial impact on dairy operations due to, for example, the increased labor costs associated with lower parlor efficiency [21]. Our findings therefore suggest that DME can negatively affect parlor efficiency and the profitability of dairy operations by increasing MMUT. 

Additionally, the average milk flow rate is a function of milk yield and the milking unit-on time, meaning that the observed differences in MAMF among cows with different DME frequencies may therefore be attributed to a decreased milk yield, increased milking unit-on time, or combination of both. We therefore believe that the observed differences in MAMF are mostly attributable to the observed association between DME and MMUT. This theory is supported by data from Bruckmaier and Blum (1996), who found that cows that did not receive premilking stimulation exhibited bimodality and increased milking duration, but no significant decrease in milk yield [4]. It is also in accordance with data from our own group showing that a short stimulation duration or lag time led to higher odds of bimodality and an increased milking unit-on time but did not negatively affect milk yield [1,2]. There still is a possibility that observed differences in MAMF could be due to decreased milk yield, as suggested by findings from previous observational studies showing an association between DME and decreased milk yield [13,14]. The authors attributed the decrease in milk yield in milking observations with DME to a higher mouthpiece chamber vacuum in cows with DME, leading to the constriction of the annular ring, which in turn can impede the milk flow between the gland and the teat cistern and decrease the amount of milk harvested during an individual milking observation [13,14]. However, because controlled experiments showing a cause–effect relationship between DME and milk yield are scarce, this possible explanation remains speculative.

In light of the association, we found that between DME and MLMF, the prevalence of DME in this study cohort may raise concerns about teat and udder health. As reported by previous researchers, teats are subjected to higher vacuum levels during periods of low or no milk flow in milking observations with DME [11,25]. Higher vacuum levels increase the risk of teat congestion [26]. These alterations of the teat circulatory system have been associated with an increased risk of intramammary infection [27], increased SCC [28], and decreased animal well-being [29]. This theory is further supported by previous work showing that an increased duration of a low milk flow rate was associated with increased risk of machine milking-induced short-term teat tissue changes (STCs) [1]. Teats affected by STC are more open and penetrable, which leads to a higher risk of new intramammary infections [30]. Though we did not directly observe higher rates of STC in this study, the increase in MLMF associated with DME shown in this study implies a risk of worsened animal health with increased DME.

Our results indicated a positive association between elevated SCC and MAMF, alongside a decrease in MMUT. This underscores the detrimental impact of subclinical mastitis on milk production, as evidenced by multiple studies [31,32]. The decline in milk yield associated with higher SCC resulted in a shorter MMUT. Furthermore, our findings revealed greater odds of DME_Q_ with higher SCC, consistent with the results reported by Zecconi et al., who documented a reduced incidence of bimodality following the administration of anti-inflammatory medication in cows with chronic mastitis [21].

Although our investigation of DME events over 7 d yielded new information on how DME affects milking performance, our study had some limitations. Firstly, we used data from only one dairy farm in upstate NY, which limits how generalizable our findings are. For example, the case definition for DME used in this study will likely need to be established on other farms with different parlor and milk flow meter systems. Secondly, herd characteristics such as the distribution of primiparous and multiparous cows within the herd, milking frequency, milking routine (including premilking stimulation), and herd performance goals are all important factors that could affect the relationship between DME and milking performance. How DME affects milking performance in farms in different areas, with different herd characteristics and milking routines, remains to be seen. With the associations our data showed between DME and decreased milking performance, we believe that cows in this study would benefit from longer premilking stimulation and a longer preparation lag time. This could lessen the prevalence of DME, but further research is required to demonstrate the effectiveness of premilking stimulation and preparation lag time in improving milking performance indices in cows with DME. Lastly, due to the observational nature of this study, a cause–effect relationship cannot be inferred.

## 5. Conclusions

The serial assessment of DME over a 1-week period in the current study revealed relationships of DME with milking performance indices, the average milk flow rate, the milking unit-on time, and the duration of a low milk flow rate in Holstein dairy cows in a large dairy herd with suboptimal premilking teat stimulation. The observed interactions between DME and cow characteristics suggest that the impact of DME on milking performance differs among cows with different parities and lactation stages. Peak lactation milk yield may serve as a proxy to estimate cows’ risk of recurrent DME. Future research is warranted to test if, for example, a modified premilking stimulation regimen has the potential to improve milking and parlor efficiency. 

## Figures and Tables

**Table 1:** Least squares means (95% confidence intervals) for mean average milk flow rate, average milking unit-on time, and average duration in low milk flow rate for hypothetical cows with 0, 7, 14, or 21 milking observations with delayed milk ejection (DME) stratified by parity and stage of lactation.

Item	DME-0	DME-7	DME-14	DME-21
Mean average milk flow rate (kg/min)				
Parity				
1	3.9 (3.8–4.0)	3.5 (3.5–3.6)	3.2 (3.1–3.2)	2.8 (2.7–2.8)
2	4.2 (4.1–4.3)	3.7 (3.7–3.8)	3.3 (3.2–3.3)	2.8 (2.7–2.9)
≥3	3.9 (3.9–4.0)	3.5 (3.5–3.5)	3.0 (3.0–3.1)	2.5 (2.5–2.6)
Stage of lactation				
1–100	4.1 (4.0–4.1)	3.6 (3.6–3.7)	3.2 (3.1–3.2)	2.7 (2.6–2.8)
101–200	4.1 (4.0–4.1)	3.7 (3.6–3.7)	3.3 (3.2–3.3)	2.9 (2.8–3.0)
>200	4.0 (3.9–4.0)	3.5 (3.4–3.5)	3.0 (3.0–3.0)	2.5 (2.5–2.6)
Mean milking unit-on time (s)				
Stage of lactation				
1–100	230 (226–233)	256 (253–258)	282 (279–284)	308 (303–312)
101–200	218 (215–222)	241 (239–243)	263 (260–265)	284 (281–288)
>200	180 (177–184)	201 (199–204)	222 (220–224)	243 (240–245)
Mean duration in low milk flow rate (s)				
Parity				
1	8 (8–9)	12 (12–12)	17 (17–18)	25 (24–26)
2	8 (8–8)	11 (11–11)	15 (15–15)	21 (20–21)
≥3	8 (8–8)	10 (10–11)	13 (12–13)	16 (15–16)
Stage of lactation				
1–100	8 (8–9)	11 (10–11)	13 (13–13)	16 (16–17)
101–200	8 (8–9)	11 (11–11)	14 (14–15)	19 (18–20)
>200	8 (7–8)	11 (11–12)	17 (17–18)	26 (25–26)

**Table 2:** Predicted probabilities (%) of delayed milk ejection (DME) for a hypothetical cow in parity 2, between 101 and 200 DIM, four lactating quarters, and a logSCC of 4.80 stratified by four different levels of peak lactation milk yields and four different levels of DME *.

Peak Lactation Milk Yield (kg/day)	DME_Q_-0	DME_Q_-7	DME_Q_-14	DME_Q_-21
<44.5	10.6	27.8	20.4	41.2
44.5–51.6	12.9	30.9	20.3	35.9
51.7–58.5	15.8	33.9	19.6	30.7
>58.5	23.5	38.3	16.9	21.3

* DME_Q_-0, 0 DME events; DME_Q_-7, 1–7 DME events; DME_Q_-14, 8–14 DME events; and DME_Q_-21, 15–21 DME events.

## Data Availability

The data presented in this study are available on request from the corresponding author.
